# Preventing Extinction of the Critically Endangered *Dactylorhiza incarnata* subsp. *ochroleuca* in Britain Using Symbiotic Seedlings for Reintroduction

**DOI:** 10.3390/microorganisms9071421

**Published:** 2021-06-30

**Authors:** Viswambharan Sarasan, Tim Pankhurst, Kazutomo Yokoya, Sridevy Sriskandarajah, Faye McDiarmid

**Affiliations:** 1 Royal Botanic Gardens, Kew, Richmond, Surrey TW9 3DS, UK; kazyokoya@gmail.com (K.Y.); mcfaye@gmail.com (F.M.); 2 Plantlife, Brewery House, 36 Milford Street, Salisbury, Wiltshire SP1 2AP, UK; Tim.Pankhurst@plantlife.org.uk (T.P.); ssridevy@hotmail.com (S.S.)

**Keywords:** reintroduction, in vitro, symbiotic, orchid mycorrhiza, fen, conservation

## Abstract

The yellow early marsh-orchid (*Dactylorhiza incarnata* subsp. *ochroleuca*) is critically endangered in the UK. Reintroduction of this threatened orchid to former haunts that have been restored is a long-term objective of this study. Identifying germination-specific mycorrhizal fungus lineages from closely related species is used as a method due to the extremely small number of plants left in the wild. A putative orchid mycorrhizal fungus of the family Tulasnellaceae, isolated from *Dactylorhiza praetermissa*, supported in vitro seed germination to produce reintroduction-ready seedlings. Reintroduced symbiotic seedlings survived over the winter months in the flooded reintroduction site (RS). The comparative soil analysis for key nutrients before reintroduction showed that phosphorus content in the RS is very low compared to the soil collected from the wild site (WS) where the last viable population exists. On the other hand, C:N ratio in the soil at the WS and RS were not significantly different. To our knowledge, this is the first-ever report on the reintroduction of symbiotic seedlings of a threatened orchid back to the wild in the UK.

## 1. Introduction

Success rates of augmenting populations in the wild worldwide are not very encouraging as rapidly increasing numbers of orchids are being included in the list of species threatened with extinction. A Scopus search in March 2021 for “orchid; mycorrhiza; reintroduction,” returned just 34 publications including review papers for the period covering the last 20 years. This number further reduced when reports on the survival or establishment of plants were included. Reintroduction is a conservation strategy often employed for threatened orchid taxa [1,2]. Reintroduction, for this study, is defined as the planting of in vitro propagated plants within their indigenous range.

Orchid biodiversity loss is a serious problem for decades in Great Britain with many species showing decline [3,4,5]. Land-use changes and issues with pollination linked to climate change are attributed as main drivers of orchid rarity [5,6]. In some taxa life span, height when in flower, and environmental factors such as soil characteristics have been shown to correlate with species loss [7,8].

**Figure 1:**
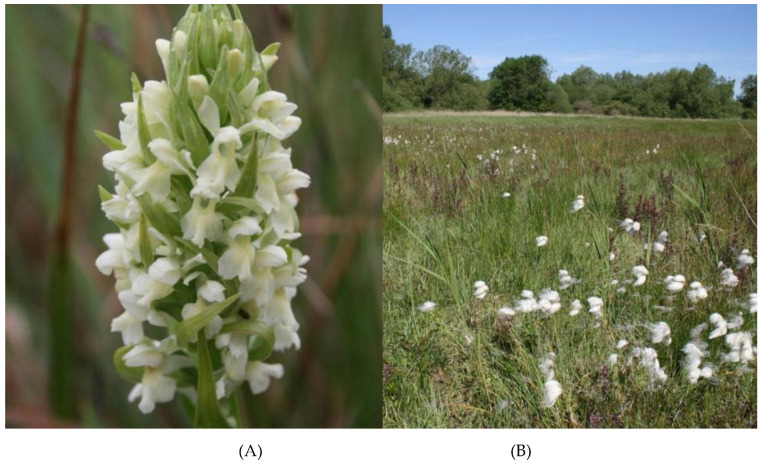
East Anglia site where the last viable population in England exists. (**A**) Flowering *Dactylorhiza incarnata* subsp. *ochroleuca*; (**B**) Fen habitat.

Yellow early marsh-orchid, *Dactylorhiza incarnata* (L.) Soó subsp. *ochroleuca* (Wüstnei ex Boll) P.F.Hunt & Summerh, is a critically endangered orchid in the UK [9] (Stroh et al. 2014). It has only ever been known from confirmed from 10 sites but occurred only transiently at five of these. It has now also been lost from one of the regular sites leaving four at which it is recorded annually. Yellow early marsh-orchid only grows in species-rich, calcareous wetland habitats of the highest order. It is normally associated with fen vegetation supplied with high quality, nutrient-poor, mineral-rich waters. The fen habitat hosts *Cladium mariscus* and *Caricion davallaniae* as abundant vegetation [10], (Figure 1). Even in seemingly ideal places, populations are limited to small areas within sites, and the limiting factors are unknown.

The complex and lengthy process of identifying seed germination-compatible mycorrhizal fungi and producing symbiotic seedlings in vitro often discourages researchers and conservationists from opting for this route. It is apparent that our progress in understanding orchids and mycorrhizal biology is not being translated to achieve more successful augmentation to support effective conservation [11]. Studies of wild populations of orchids have shown that the putative mycorrhizal fungi capable of supporting germination are, at least in some cases, a subset of those that associate with the adult plants [12,13]. In other cases, a different set of mycorrhizal fungus altogether may be responsible for germination [14], or this can vary between different habitats [15,16]. The use of a closely related species as a mycorrhizal donor for a species that is on the brink of extinction is a potentially powerful approach; investigating taxa that are closely related to rare orchid species can help to effectively propagate seedlings of the recipient orchid for reintroduction. Experimental reintroductions require a large quantity of material to be propagated from seed, but the isolation of the necessary fungi from the roots of wild plants is not an option because of the likely damage to the small, very vulnerable, population.

Propagation techniques have therefore been developed using fungi from closely related species in the present study. Investigating whether putative mycorrhizal fungi from closely related species can be used to germinate seeds of threatened orchids to augment populations in the wild is the main objective of this study. The efficacy of producing symbiotic seedlings suitable for growing in identified reintroduction sites within the native range and translocation outside of its native range is discussed.

## 2. Materials and Methods

### 2.1. Soil Analysis

Soil samples were collected from three study sites: wild site (WS), reintroduction site (RS), and mycorrhiza origin site (MS). WS is where *D. incarnata* subsp. *ochroleuca* wild plants exist; RS is the site where in vitro seedlings were introduced; MS is the site from where the mycorrhizal host *D. praetermissa* was collected. Three soil samples from WS, six samples from RS, and two samples from MS were collected and analyzed for pH, N, P, K, Mg, and soil organic matter (SOM). C:N ratio was calculated as SOM/total N/1.72. An unpaired *t*-test with Welch’s correction was used to analyze the data.

### 2.2. Population Count and Seed Collecting

The study sites in East Anglia, England with remaining populations of *D*. *incarnata* subsp. *ochroleuca* are managed as nature reserves by the Suffolk Wildlife Trust. The locations of individual plants have been recorded at all known sites using a handheld navigation device (Garmin eTrex 32×, Garmin Europe Ltd, Hampshire, UK) with variable accuracy of 3 to 5 m, and total numbers estimated using that data. Currently, sites are visited several times and individual plants are tracked through flowering to take account of a decline in visibility as flowering plants can be eaten off by deer, mainly Reeves’ Muntjac (*Muntiacus reevesi*), an alien species in the UK. This approach has however only recently been applied and counts to 2018 are accordingly not fully reliable.

**Figure 2:**
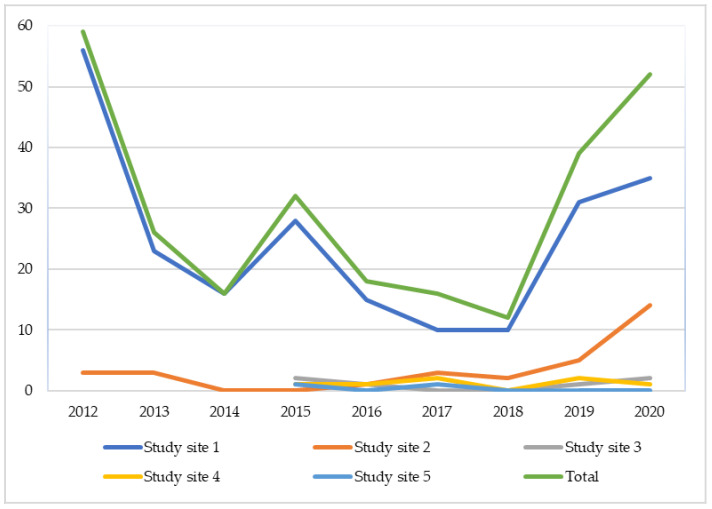
Counts of *Dactylorhiza incarnata* subsp. *ochroleuca* recorded over nine years in England in five study sites.

There are two populations at the Suffolk study site 1 (population A and B) and only one of these do plants reliably appear which is Population A. Seeds were collected from Population A from the study site 1 (Figure 2) where the plants were identified during the flowering season. Low incidence of seed set (due to grazing) requires some flowering plants to be protected by covering with individual chicken-wire cages, pinned to the substrate with short bamboo poles. These plants were then revisited, and seed capsules were gathered. Mature seed capsules were collected in the second week of August for symbiotic germination. Seeds were dried, transferred to vials, and stored at 4 °C.

### 2.3. Identification of Compatible Mycorrhizal Fungus for Seed Germination

Since the identification of putative mycorrhizal fungi from remaining plants of *D. incarnata* subsp. *ochroleuca* in the wild was not feasible, putative mycorrhizal fungi from closely related and other orchids were tested for seed germination. Root samples were collected from Chafford Gorges, an Essex Wildlife Trust site (the mycorrhiza origin site, MS) during summer. Root samples were collected from a total of five plants from two populations of *D. praetermissa*, *D. fuchsii,* and *N. ovata*. The plants were growing in a wet chalky site. Roots were sliced thinly, and pelotons were extracted and cultured as described by [4]. Hyphae appearing from pelotons were individually isolated for six weeks or until the mycelia became indistinguishable. Sporulating fungi were discarded until putative mycorrhizal fungi and non-sporulating fungi, such as the dark septate fungi, remained. Subculturing was repeated to maintain pure cultures. ITS sequencing of the internal transcribed spacer region (ITS) of the rRNA gene was done as described by [17]. Briefly, DNA was extracted from hyphae using Sigma Extract-N-Amp™ Plant Tissue PCR Kit (Sigma Aldrich, St. Louis, MO, USA). PCR amplification of the ITS region using primer ITS1F with ITS4, and ITS1 with ITS4-tul [18,19,20,21] was followed by Sanger sequencing using the same forward and reverse primers. Sequences were compared to GenBank database sequences using BLAST (National Center for Biotechnology Information, Bethesda, MD, USA). Sequences that matched *Rhizoctonia*-like fungi were aligned and grouped into operational taxonomic units (OTUs) based on a conservative similarity threshold of 95%. Representative sequences of each OTU were deposited in GenBank (Supplementary Materials). Orchid mycorrhizal fungal isolates (OMFs) were cryopreserved at RBG Kew.

### 2.4. In Vitro Seed Germination

Seeds were tested for viability using 2,3,5 triphenyl-2H-tetrazolium chloride (TTC) (VWR Leicestershire, UK) as described by [22]. For symbiotic germination, seeds were surface sterilized with 0.5% (*w/v*) sodium dichloroisocyanurate (Sigma Aldrich, St. Louis, MO, USA) for 40 min and sown on oatmeal agar (OMA) [23] in 5 cm Petri dishes. Culture media (pH adjusted to 5.8 and 8.5) were autoclaved at 121 °C for 15 min.

Seeds were inoculated with fungal isolates by placing approximately 0.3 cm cube of agar containing the mycelium onto the OMA medium (one OMF isolate per dish) after sowing the seeds. Control replicates of seeds sown on OMF-free OMA medium were also made. Each Petri dish was sealed with Parafilm M^®^ (Pechiney Plastic Packaging, Menasha, WI, USA) and incubated in dark and seedlings at 22 °C ± 2 °C and 16/8 h photoperiod under cool white, fluorescent light at 20 µmol m^−2^ s^−1^. Germinating seeds were scored every 4 weeks for 6 months using the method of [24] followed by the emergence of the first leaf as part of seedling development. Ten replicate plates were set up for each treatment with a minimum of 60 embryo-bearing seeds per replicate.

OMFs derived from Chafford Hundred chalky calcareous areas were selected due to the good number of orchid populations found at the site, predominantly *Dactylorhiza* species. Among those tested were putative mycorrhizal fungal isolates from *Dactylorhiza praetermissa* (Tul1) and *D. fuchsii* (Tul2), see Table 1. Since the isolates of each OTU differed slightly in their growth some of the isolates from each OTU were tried. After the initial trial, Tul2 was eliminated from further trials as they failed to produce good quality seedlings. Further tests were conducted with Tul1(03DP009) isolate from *D*. *praetermissa* as this was identified as the compatible mycorrhizal fungus isolate. The germination studies repeated three times with different variants of Tul1(03DP009) to study the potency of the compatible mycorrhizal fungus after isolating the pelotons from symbiotic seedlings grown in vitro. Five variants were identified and tested for germination and seedling development using different seed accessions and at pH 5.8 and 8.5 (to get a pH value of around 8 after autoclaving as this is close to wild soil pH), see Supplementary Materials.

### 2.5. Transfer of Seedlings to Preweaning Medium

The very early-stage seedlings of 5 mm long were transferred to two media to compare their establishment rate. Vermiculite mixed with paper pulp (1:1) supplemented with full-strength Hoagland’s medium [25], and a 1:1:1 mix of vermiculite, paper pulp, and soil from WS (mix of all three soil samples), were tested for this trial. Data on leaf emergence and growth were collected over a period of six months. Plants were subsequently grown on medium with WS soil in honey jars for one year before reintroduction to RS, near WS. Soil collected from WS was stored untreated at 4 °C and used within a week without autoclaving.

### 2.6. Resilience of Seedlings at Reduced Moisture Content

Single seedlings raised symbiotically were grown in 30 mL dilu vials with varying amounts of moisture. They were grown in a supporting mix made of vermiculite and paper pulp over six months and no fresh medium was added during this period of growth. The samples were split as wet (10 cm^3^ plug with 4 mL of moisture), moist (10 cm^3^ plug with 2 mL of moisture), and dry plugs (10 cm^3^ plug with 0.5 mL of moisture). Data of fresh new leaves developed and/or dormancy was recorded.

### 2.7. Reintroduction of Symbiotic Seedlings

**Figure 3:**
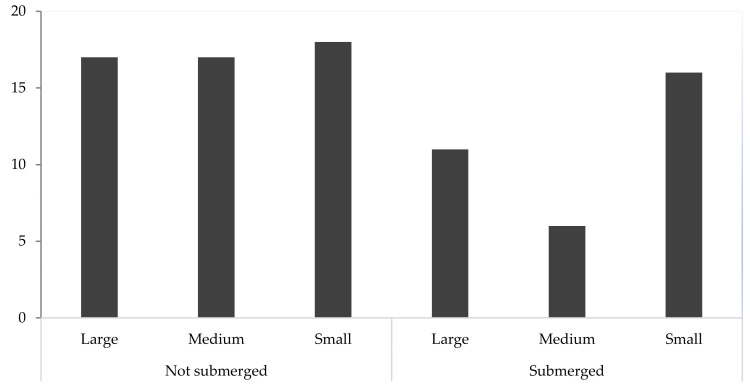
Reintroduction plan showing samples (2-3 seedlings/sample) of different size seedlings (small, medium, and large) of *Dactylorhiza incarnata* subsp. *ochroleuca* planted in the wild site. Seedlings were either submerged or not submerged at the time of planting.

Seedlings of different sizes (small <2cm shoot, medium 2cm to 4cm, large >4cm) were reintroduced to the flooded fen 10 miles away from the wild site in September 2020 (Figure 3). In most years, this site floods, and mire vegetation is submerged most of the year. Data on survival were collected at the end of April 2021 to assess early survivorship.

## 3. Results

### 3.1. Soil Analysis

**Figure 4:**
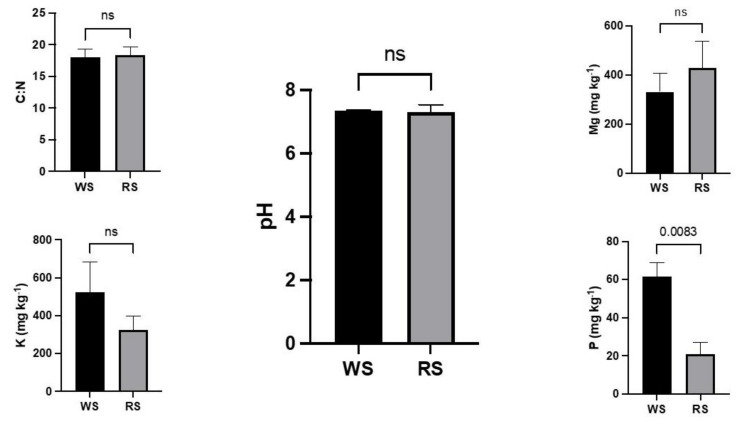
Results of *Dactylorhiza incarnata* subsp. *ochroleuca* wild site soil showing C:N, pH, and other minerals in mg/Kg of soil. Data analyzed following an unpaired *t*-test with Welch’s correction.

RS had populations of yellow early marsh-orchid in the past. There was no significant difference between pH values of soil at WS and RS (Figure 4). The WS soil had nearly three times high average P content compared to RS (*p* = 0.0083). On the other hand, C:N ratio, K and Mg content in the soil at the WS and RS were not significantly different.

Unlike the Suffolk study sites, (wild site, WS, and reintroduction site, RS), the Chafford Hundred site in Essex (MS) is not a fen but instead is a restored chalk mine, a successful habitat for orchids such as *Anacamptis pyramidalis*, *Dactylorhiza praetermissa*, *D. fuchsii*, *Epipactis phyllanthes*, *Neottia nidus-avis*, *Ophrys apifera*, *Neottia ovata,* and *Orchis anthropophora*.

### 3.2. Population Count and Seed Collecting

Plotting the records on a map enables a proportion of individual plants to be tracked and an estimate of the total population to be built up over the course of a month. When the plant does occur, it does so in small numbers, the most ever recorded at one site being 56 (Figure 2, orchid counts at known sites). However, counts are probably under-estimates as (i) it is not possible to confirm the identity of the plant unless it is flowering and (ii) flower heads are commonly browsed off by deer soon after opening.

The total number declined from around sixty individuals to just 12 in 2018 but recovered to 52 plants by 2020. The species appeared for the first time at three of these sites during this period. There are two populations at study site 1 (population A and B) and only one of these do plants reliably appear which is Population A. Seeds were collected from Population A from the study site 1 (Figure 2) where the plants were identified during the flowering season and caged for seed collecting after few weeks.

### 3.3. Identification of Compatible Mycorrhizal Fungus for Seed Germination

More than 60% of pelotons isolated from root sections developed into fungal cultures. Fungal cultures that displayed morphological characteristics such as monolioid cells were prioritized for serial culturing to get pure cultures for identification. Two different putative mycorrhizal fungal OTUs were identified from *D. praetermissa* and *D. fuchsii* (Supplementary Materials- Sequences of Tul1 and Tul2). Both were OTUs of Tulasnellaceae clade which were assessed for symbiotic seed germination. Although all isolates were used in the initial preliminary trial to identify compatible seed germination-specific fungi, only one OTU, Tul1, (closest Genbank match JX649077) stimulated seed germination.

### 3.4. In Vitro Seed Germination

**Figure 5:**
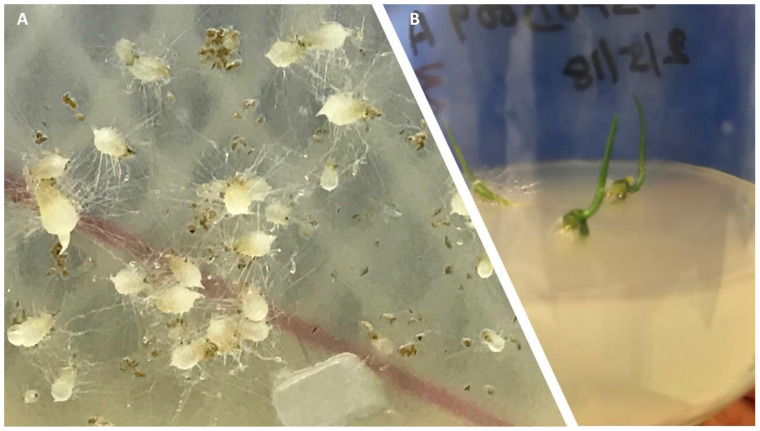
Symbiotic seed germination of *D. incarnata* subsp. *ochroleuca* with Tul1 isolate 03DP009 (closest match JX649077): (**A**) protocorms and developing seedlings (**B**) seedlings.

**Figure 6:**
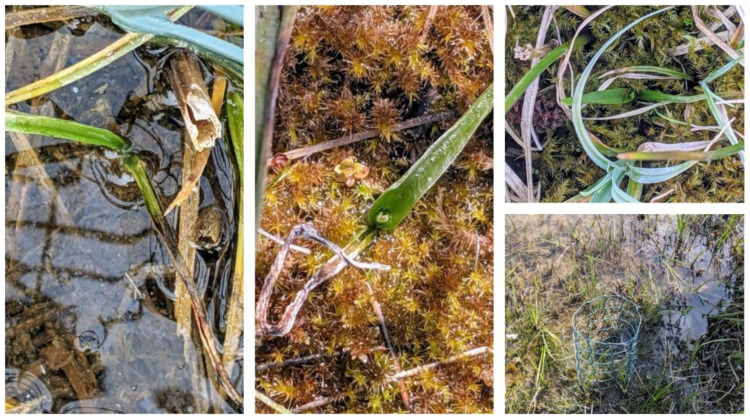
*Dactylorhiza incarnata* subsp. *ochroleuca* seedlings of different sizes (small, medium, and large) established after seven months of reintroduction to the flooded fen.

After preliminary studies using putative mycorrhizal fungi from *Dactylorhiza praetermissa* and *Dactylorhiza fuchsii* OTUs (Tul1 and Tul2, Table S1) were found to be the compatible fungus for protocorm development (Figure 6). Protocorm development was completed over eight weeks while progression to seedlings occurred after the following 8–12 weeks (Figure 5, Figure S1). There were differences among seed accessions due to variations in the viability of seeds, but this was not statistically significant. Seed viability varied between capsules within a plant and among plants. Similarly, there was no significant difference in germination between the two media pH tried. Different variants of the Tul1 03DP009, based on their growth characteristics rather than sequence variations, when used for germination results showed no significant differences (Table S2).

Smaller protocorms, about 2 mm in diameter, had fewer rhizoids and failed to progress to seedlings. Generally, shoot primordia were visible after eight weeks and further growth of seedlings was improved when they were transferred from darkness to diffuse light (16/8 light/dark cycles) at 22 °C. Tul1 supported full seedling development while Tul2 failed to produce any good quality seedling. Tul1 isolate 03DP009 was found to be the compatible OMF that consistently produced good quality seedlings. Seedlings raised in WS soil without any supplemented nutrient medium performed better than on medium without soil with Hoagland’s medium, producing 30% greater growth over six months.

### 3.5. Transfer of Seedlings to Preweaning Medium

Seedlings grown on a 1:1:1 mix of vermiculite, paper pulp and soil from the wild site showed 100% survival and better growth compared to the medium containing vermiculite mixed with paper pulp (1:1) supplemented with full-strength Hoagland’s medium. The growth of the seedlings transferred from the mixes (with or without wild soil) was not significantly different. There was no adverse effect on seedlings when they were grown on nonsterile soil from the wild site.

### 3.6. Resilience of Seedlings at Reduced Moisture Content

All seedlings grew but moist plugs supported the best growth followed by the wet plug. In dry plugs, seedlings grew initially for eight weeks and later became dormant. Seedlings raised symbiotically grew in wet, moist, and dry plugs, made of vermiculite and paper pulp over six months.

### 3.7. Reintroduction of Symbiotic Seedlings

Seedlings of all sizes, either submerged or not submerged at the time of planting, survived over the winter of 2020–2021 (Figure 6). After seven months of planting 42% of seedlings showing growth after the winter months. Seedlings were not observed in areas where the water level is more than 10 cm in depth. Seedlings were visible in association with seasonally emerging vegetation characteristic of the reintroduction target area, typified by *Carex elata, Schoenus nigricans, Phragmites australis, Eleocharis quinqueflora* and *Juncus subnodulosus,* mosses *Campylium stellatum* and *Calliergonella cuspidata* and, in inundated areas, the stoneworts *Chara vulgaris, C. aculeolata,* and *C. hispida.*

**Figure 7:**
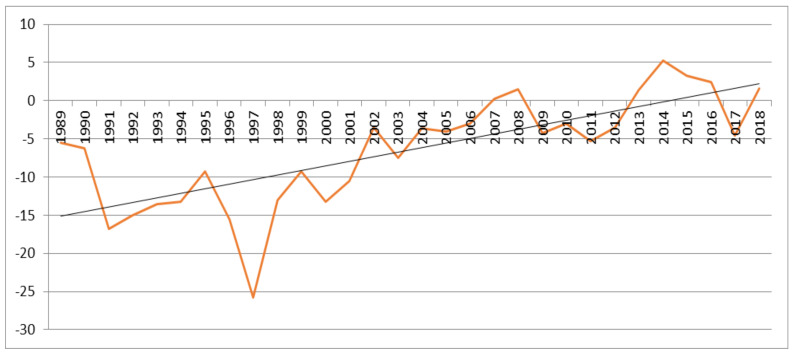
Average height of water table in relation to ground level in March/April in cm in Chippenham Fen National Nature Reserve (NNR), a former site.

The average height of the water table in relation to ground level based on data from Chippenham Fen NNR shows increased wetness in the last 20 years (Figure 7). During this period population size also declined.

## 4. Discussion

Monitoring methods for this taxon are constrained by the fact that it can only reliably be identified when in flower. This occurs between mid-May and mid-June. They are also hampered by grazing, the plant being particularly attractive to deer when in flower. Therefore, plants in flower one week may have been eaten off a week later but replaced by new plants. Accurate population assessment, therefore, involves multiple surveys across the flowering window. However, more accurate (i.e., sub-meter) plotting would be advantageous and facilitate better tracking. Gathering seeds to germinate is hampered by the same circumstances. It may be those sites at which the plant appears sporadically that may have plants every year but not in a flowering condition. Despite caveats associated with the population estimates, the disappearance from Chippenham Fen is unequivocal as the plants were last seen in 2004 seemingly following a long slow decline [26].

For long-lived plants such as the Orchidaceae estimates of viable population size vary from 50 to 200 individuals [27]. Because of the small number of individuals of *D. incarnata* subsp. *ochroleuca* left in the wild, other sources for germination specific mycorrhizal fungus were explored. A site more than 100 miles, a restored chalk quarry, which is a successful site for many orchids including southern marsh orchid (*D*. *pratermissa*) was chosen. As this site is hosting a diverse range of orchids seed-germination-compatible *Tulasnella* OTU from southern marsh orchid (from a chalky wet habitat) was tested among many other isolates of putative mycorrhizal fungi for yellow early marsh. The lineage of Tulasnellaceae, a common mycorrhizal fungus in orchids shows generalist behavior as reported in many *Dactylorhiza* and other terrestrial orchids [28,29,30,31,32].

Some species of *Dactylorhiza* are associated with a wide range of Tulasnellaceae OTUs, of which some are distributed widely and common [33]. In three terrestrial orchids, it is demonstrated that one *Tulasnella* strain exhibited high compatibility across the three distantly related host species showing the generalist nature of this lineage of mycorrhizal fungus [29]. Studies in several species of the terrestrial genus of *Cynorkis* from Madagascar and found that most of those species recruited fungi of *Tulasnella* lineage at various growth phases and under different soil conditions [31]. In the current study the donor orchid, *D. praetermissa* was from a moist chalky site while the recipient orchid was from a chalky fen habitat. Although orchid mycorrhizal fungi are characterized by having a broad geographic distribution their occurrence is influenced by specific habitat conditions [33] which means understanding related orchid species in similar soil conditions may help understand the fungal diversity and similarities.

The result of this study shows the only remaining population is in a nutrient-rich habitat compared to several other reports where orchids are found in nutrient-poor habitats. In *Cephalanthera rubra* [4] and *Cynorkis* [31] it is found that elevated P content leads to lower mycorrhizal diversity. Differences in mycorrhizal diversity among sites were driven by differences in soil P and N content [34], the authors argue that higher soil nutrient availability promotes specialization in orchid–mycorrhizal associations for soils with high N availability. The preliminary results on the survival of seedlings during the flooded winter months show that the reintroduction site support growth although P content is significantly low compared to the wild side. Total N content and C:N ratio was not significantly different in both WS and RS. More detailed studies are essential to understand why populations of this orchid declined in many fens with similar soil conditions. Additionally, detailed studies are essential to understand the reasons why seedling recruitment is not occurring at wild sites for decades.

It has been shown that understanding the mycorrhizal diversity and density in the reintroduction site is also key to successful population establishment [35]. Testing sites suitable for reintroduction/translocation of rare orchids with symbiotic seedlings offer opportunities. Seedlings raised symbiotically can be planted in historical sites where the orchids have been growing in the past but were lost lately. Knowledge of fungal diversity and density in the receiver site must be studied in detail especially if the site has never hosted the species identified for reintroduction/translocation. The introduced orchids will contain effective fungi, but they can also run the risk that the site may not be suitable for fungus and/or plant in the long term [1,16]. A review of successful reintroduction projects in orchids showed that mycorrhizal presence to be one of two key factors in the continued resilience of reintroduced orchid populations (the other factor being pollinator presence) [1]. They therefore also recommend symbiotic propagation.

Seed baiting performed in the past failed to retrieve any protocorms of *Dactylorhiza incarnata* subsp. *ochroleuca* from WS. As an alternative approach to identifying a compatible mycorrhizal fungus, a *Tulasnella* culture isolated from mature *D. praetermissa* plants was tested for symbiotic germination and seedling growth. Despite having isolated from MS which is located more than 100 miles away from WS where *Dactylorhiza incarnata* subsp. *ochroleuca* is found, it was shown that a mycorrhizal fungus from a closely related species from a different habitat could be used in reintroduction projects using symbiotic seedlings. Both sites had similar soil pH, which may help the establishment of symbiotic plants in the wild habitat. However, flowering, natural seedling recruitment, and resilience of translocated populations over a longer period must be monitored to understand the full feasibility of this method.

Putative mycorrhizal fungi from protocorms [36] or seedlings [37,38] serve as reliable symbionts for seed germination. In some cases, the orchid seeds can only be germinated by a specific lineage of the mycorrhizal fungus from protocorms [35]. The current study used fungi available from plants at different growth phases and found that *Tulsanella* OTU from mature phase *D. praetermissa* was a germination-compatible fungus. This has been proved as a successful approach in identifying putative mycorrhizal fungi in orchids from Madagascar [17,24,31,32,39]. Putative mycorrhizal fungus isolate from seedlings was more efficient at supporting seed germination compared to those isolated from adult roots in *Dendrobium exile* [38,40] suggested that the fungi used in conservation must be limited to mycorrhizal fungi acquired from the same or nearby populations. In this case, this was not feasible but compatible fungus from a closely related species was used successfully.

The reintroduction site, RS, identified as a suitable site is a fen ecosystem where the water table is at or close to ground level for much of the year. *D. incarnata* is intolerant of prolonged inundation with the water table in spring-fed fens being relatively stable [41]. But our studies using seedlings showed a different picture as the symbiotic seedlings were resilient to tolerate extreme conditions of wetness. Most of them survived over a long period during the winter months and even into spring after reintroduction in September. Over a period of more than seven months as East Anglia endured one of the wettest winters on record. As there is very little drainage from the fen the seedlings were completely inundated throughout this period. Seedlings still inundated were not visible to assess as the water level is too high to see them (about 30% of the seedlings). These seedlings may start growing during the late spring and summer months when the water level receded. Symbiotic seedlings displayed resilience when they were grown in growth chambers in moist and dry conditions. As the plants left in the wild are growing in wet conditions in a fen habitat symbiotic seedling survival at both extreme shows the resilience of these propagules for scaling up and larger studies to test reintroduction/assisted colonization in the future. Additionally, long-term studies underpinning soil conditions and recruitment of orchid mycorrhizal fungi by the reintroduced seedlings will help understand the resilience of the reintroduced populations.

The conservation strategy for orchids is focused on developing insight into the ecological requirements of the taxon and understanding the reasons behind its decline. The decline and disappearance of this orchid in a fen habitat have at some sites, been contemporaneous with hydrological changes. The fen site selected for the reintroduction was specifically chosen as it is known to have a stable water table learned through hydrological monitoring. Natural England and the Suffolk Wildlife Trust have hydrological monitoring facilities installed in all the fens earmarked for future reintroduction. Climate change can exacerbate the decline of threatened taxa as an interplay between biotic (mycorrhiza) and abiotic factors (hydrology, pH, nutrients) can make previously suitable sites less hospitable. The effects of temperature and rainfall on mycorrhiza have been studied [42,43]. It is critical to assess how abiotic factors influence the dynamics of both the orchid and its mycorrhizal lineages, particularly for species that are threatened with extinction.

In a study for the diversity of orchid mycorrhizal fungi as a function of the water availability of the habitat Tulasnellaceae group of fungi did not favor being flooded with water but had good tolerance of dryness [44]. Our studies under controlled conditions show the symbiotic seedlings tolerated dryness. Survival of reintroduced symbiotic seedlings which were completely inundated showed *D. incarnata* subsp. *ochroleuca* can tolerate long periods of inundation with *Tulasnella* in their roots. This means the symbiotic seedlings can tolerate both dry and flooded conditions.

Greater OMF abundance in the soil is considered as one of the critical factors that render the wild population more able to withstand the influences of climate change [35]. Additionally, identifying the mycorrhizal fungal diversity and abundance using the qPCR method offers quick and reliable information on the suitability of the proposed reintroduction/translocation site. Understanding the symbiotic behavior of orchids to produce seedlings that are suitable for augmenting in the wild is an important aspect in addition to protecting plants in the wild as studied in orchids of Madagascar [26,36]. Following the successful establishment of plants after reintroduction applying different tools such as qPCR to identify fungi at different seasons will help understand the fungal recruitment behavior and resilience of plants. The use of both culture-dependent and culture-independent studies is essential to understand the full suite of mycorrhizal fungi [45].

## 5. Conclusions

Augmenting populations of fragmented and critically endangered orchids is fraught with many challenges. Identifying germination specific OMF from a closely related species was found to be useful in producing resilient symbiotic seedlings suitable for preliminary reintroduction trials. As the remaining wild population is very small assessment for fungal diversity and abundance in roots of the wild population is not practical. Further studies to assess wild soil and selected sites for reintroduction for mycorrhizal fungal diversity and abundance will help understand the right conditions for the successful establishment of plants. Such assessments underpinned by soil and hydrological data will further help the process of large-scale reintroduction/translocation studies. Additionally, these types of projects require long-term monitoring of plants in the wild to assess the benefits. Although the results in the study are from limited preliminary studies in vitro raised symbiotic seedlings displayed tolerance to sub-optimal moisture content and inundation in the wild for prolonged periods of time. This preliminary information warrants detailed investigation using larger samples of symbiotic seedlings to understand its ability to establish outside the natural range. Understanding the right sites at previous habitats and non-native ranges will provide valuable information to support the conservation of this critically endangered orchid in the age of climate change.

## Figures and Tables

**Table 1:** Source of mycorrhizal fungi, closest match, and isolates used for preliminary seed germination trials of *Dactylorhiza incarnata* subsp. *ochroleuca*.

Orchid Genotype	Closest Match	%ID	Identity	Isolates
*Dactylorhiza praetermissa*	JX649077	96.7	Tulasnellaceae Tul1	03DPR003 03DPR004 03DPR005 03DPR006 03DPR007 03DPR008 03DPR009 03DPR011
*Dactylorhiza fuchsii*	JX649080	93.5	Tulasnellaceae Tul2	01DFU012 01DFU030 01DFU032 01DFU034 01DFU042 01DFU043 01DFU052 01DFU055

## Data Availability

Not applicable.

## Supplementary Materials

The following are available online at https://www.mdpi.com/article/10.3390/microorganisms9071421/s1, Figure S1: Protocorm and seedling percentages, Table S1: DNA sequences of Tul1 and Tul2, Table S2: Seed germination using different variants of 03DPR009 at two pH levels.
