# Supplementary material for: Preventing Extinction of the Critically Endangered Dactylorhiza incarnata subsp. ochroleuca in Britain Using Symbiotic Seedlings for Reintroduction
Source: Microorganisms. 2021 Jun 30;9(7):1421. doi: 10.3390/microorganisms9071421 (PMC8303340; doi:10.3390/microorganisms9071421)
Supplement: Supplementary file 1 [file microorganisms-09-01421-s001.zip › microorganisms-1269528-supplementary.pdf]

Supplemental Files

Table S1: DNA sequences of Tul1 and Tul2

Sequences o Tul1	*Tul1-Closest match JX649077, %ID=96.74 Tulasnellaceae*	GGCGGAGCCGTTCGAACCATGCGTCATGCGTCGTGTTATCCCTCTCGGGACACACGTAAACCCTTACATGCACAGAGTATGCCTCT<br>TACGAAAACAAACAAAGAAATCACAACCGGTAGCGTTGGATCCCTTGGCACGTTGATCGATGAAGACCGTTGCAAACTGCGATAAGT<br>GGTGTGATGCGCAAGTCTAAAACTTATAAGTGAATCATCGAATCGTTGAACGCACGACACCGGCTCTATCCGAGCAGGTATGCCCCT<br>TTGAGCGCCATTATGCCTTCGGAGCATCCGGATTTCNGTCCGTGATGCCCGGGTTCGAGGTCCACCGTGCGTCCACGCAGGCGTTC<br>CTTCAGATGCATTGGAAGGGAAGAGGCCACGCCGGAACCCATCCGTGTCATGAGACATGCACGGAAAGGACCGCAGCGCCGAAGCC<br>CGGGACGTGTCCGCAGCGAGACCGTCCAGACNCACCTTCGCGGTGAAAACCATNGAATGTGGCCTCACTGGGGTAAGACTACCCGCT<br>AGACTTAAGCATATTAATCAGCGGAGGAAAAGAAACTAACTAGGATTCCCTCAGTAACTGCGAGTGAAGCGGGATGAGCTCAATGTGTGAATCTGGCGG
Sequences o Tul1	*Tul2- Closest match JX649080, %ID=93.52 Tulasnellaceae*	CTGACTGGTATCTGTTGGTCTTTCGCGTGATATATGTTAGTCTCCGTTGTCTACCCCAATGAGGTCATGCCTTGCTAGTCCGCGATCCCA<br>GGACCGGACTTGGTGACCAGCCCAACGGCGGTCAAGCACGTCTCAACTCCAGAGGACTCCCGCTCAGGCATTAGAGTACCCATCAGGC<br>GATCGGCGCGACGCAGCTGAGAAACACGATCCAAAGACCGAGGACTCCGAACTCGGGTCTTCCACAAGGAAAGACTCCCGAAGAATAAA<br>TGACGCTCAAGGGGCATACCGCAGCCGGATTAGGGCGCGGTGCAATGCGTTCAACAACTCGATGATTCACGTATAAGTGGTGGACTTGCG<br>CATCACATCATTTATCGCAATTTGCAACGGTCTTCATCGAATGACGTGCCAAGGGATCCAGCGCTGCCAGTTGTAAAAGTTTACAACTGTTA<br>GACTCACAACGCGGAACGATCTTTAACGTGTGCCTCGAACAGAGGTAACACAGCGACCGCTTTACCTCGTCAGAGGGTCGTTTATCCTCGA<br>CCAGAGCCCTTAACGTCCCAGGACGACGGAACAGAACGTCAAGACGATACTATGATCTTCCGCAT

Table S2: Seed germination using different variants of 03DPR009 at two pH levels.

Seeds tested using different isolates of the		
**Variants of 03DPR009**		
Fungal Isolate	pH	Avg. % Germ
None	5.50	16.1
None	8.5	20.1
DP009A	5.50	88.3
DP009A	8.50	83.9
DP009S1	5.50	44.4
DP009S1	8.50	43.6
DP009S4	5.50	56.8
DP009S4	8.50	7.9
DP009S5	5.50	12.3
DP009S5	8.50	85.8
DP009S3	5.50	58.8
DP009S3	8.50	82.5
None	5.50	12.7
None	8.50	17.3
DP009A	5.50	19.5
DP009A	8.50	6.1
DP009S4	5.50	48.7
DP009S4	8.50	46.3
DP009S3	5.50	24.1
DP009S3	8.50	34.1
DP009S1	5.50	51.2
DP009S1	8.50	34.7
DP009S5	5.50	57.7
DP009S5	8.50	6.7
None	5.50	9.4
None	8.50	11.9
DP009A	5.50	38.7
DP009A	8.50	23.8
DP009S5	5.50	12.4
DP009S5	8.50	39.5
DP009S3	5.50	45.7
DP009S3	8.50	32.8
DP009S4	5.50	35.3
DP009S4	8.50	34
DP009S1	5.50	40.4
DP009S1	8.50	31.9
None	5.50	10.1
None	8.50	13.8
DP009S4	5.50	23.6
DP009S4	8.50	75
DP009S1	5.50	68.9
DP009S1	8.50	77.7
DP009A	5.50	40.4
DP009A	8.50	61.2
DP009S5	5.50	35.9
DP009S5	8.50	71.5
DP009S3	5.50	31.4
DP009S3	8.50	55.4

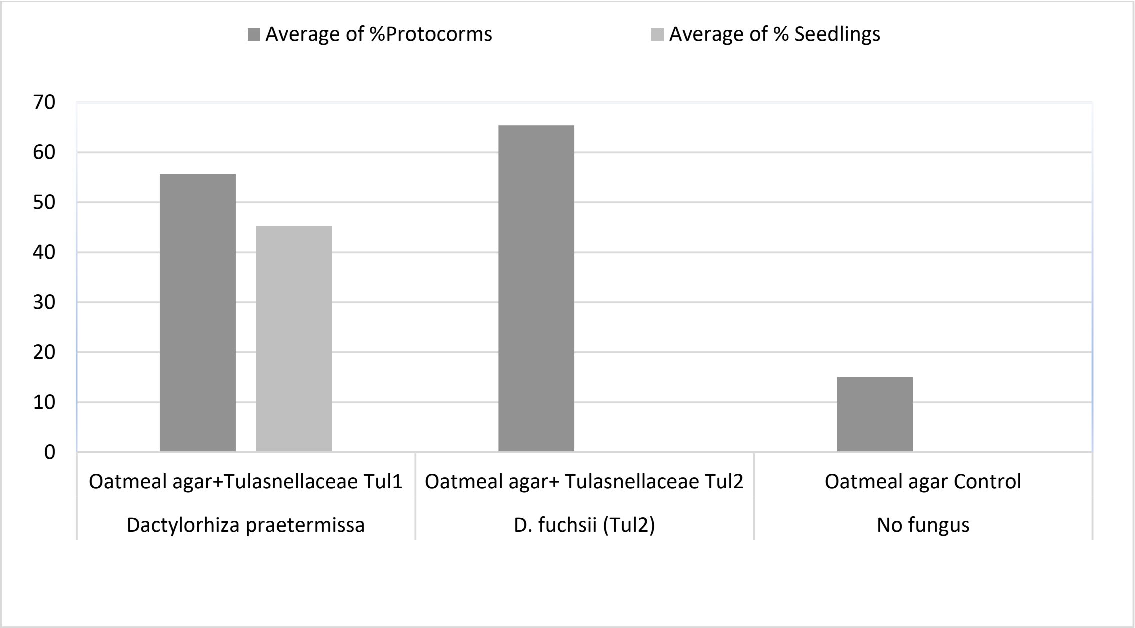

Figure S1, Seed germination: Percentage of seeds germinated to protocorms and seedlings with Tul1, Tul2 and without mycorrhizal fungus (control).

Supplemental Table. Average percentage germination when seeds co-cultured with different variants of Tul1 03DP009 fungus.
